# De novo prediction of functional effects of genetic variants from DNA sequences based on context-specific molecular information

**DOI:** 10.3389/fsysb.2024.1402664

**Published:** 2024-06-03

**Authors:** Jiaxin Yang, Sikta Das Adhikari, Hao Wang, Binbin Huang, Wenjie Qi, Yuehua Cui, Jianrong Wang

**Affiliations:** ^1^ Department of Computational Mathematics, Science and Engineering, Michigan State University, East Lansing, MI, United States; ^2^ Department of Statistics and Probability, Michigan State University, East Lansing, MI, United States; ^3^ Department of Biomedical Engineering, Michigan State University, East Lansing, MI, United States

**Keywords:** genetic variants, deep learning, DNA sequence, disease genetics, systems genetics, cellular context specificity, foundation models

## Abstract

Deciphering the functional effects of noncoding genetic variants stands as a fundamental challenge in human genetics. Traditional approaches, such as Genome-Wide Association Studies (GWAS), Transcriptome-Wide Association Studies (TWAS), and Quantitative Trait Loci (QTL) studies, are constrained by obscured the underlying molecular-level mechanisms, making it challenging to unravel the genetic basis of complex traits. The advent of Next-Generation Sequencing (NGS) technologies has enabled context-specific genome-wide measurements, encompassing gene expression, chromatin accessibility, epigenetic marks, and transcription factor binding sites, to be obtained across diverse cell types and tissues, paving the way for decoding genetic variation effects directly from DNA sequences only. The *de novo* predictions of functional effects are pivotal for enhancing our comprehension of transcriptional regulation and its disruptions caused by the plethora of noncoding genetic variants linked to human diseases and traits. This review provides a systematic overview of the state-of-the-art models and algorithms for genetic variant effect predictions, including traditional sequence-based models, Deep Learning models, and the cutting-edge Foundation Models. It delves into the ongoing challenges and prospective directions, presenting an in-depth perspective on contemporary developments in this domain.

## Introduction

Genetic variants have emerged as pivotal factors in the etiology of severe human diseases (Klein et al., 2005). Therefore, quantitative and systems-level understandings of the relationship between human diseases and genetic variants are critical in precision medicine and clinical care. Over the past decades, the Genome-wide Association Study (GWAS) (Hirschhorn and Daly, 2005; Visscher et al., 2012) has revolutionized the field of complex disease genetics, in which millions of single-nucleotide polymorphisms (SNPs) of individuals are tested to identify significant genotype-phenotype associations. However, GWAS grapples with two pronounced limitations that have spurred the quest for advanced methodologies (Tam et al., 2019). Firstly, it often limited by low statistical power, mainly stemming from the constraints imposed by limited sample sizes and the arduous multi-testing demands. Secondly, the causal relationships between specific genetic variants and diseases remain obscured, partly owing to the ambiguity induced by Linkage Disequilibrium (LD) (Bulik-Sullivan et al., 2015) and the paucity of insights into the underlying molecular mechanisms. Traditionally, human disease genetics research has centered around SNPs located in protein coding regions, a mere 1.2% of the human genome (Visscher et al., 2012). Next-generation Sequencing (NGS) (Buermans and den Dunnen, 2014) technologies like RNA-seq, DNase-seq, and ChIP-seq (Luo et al., 2020) have empowered researchers to measure gene expression, chromatin accessibility, and transcription factor (TF) binding genome-wide. This advance fuels an exploration of the vast non-coding genome and gives the potential to analyze the effect of genetic variants on nearby local regions.

**FIGURE 1:**
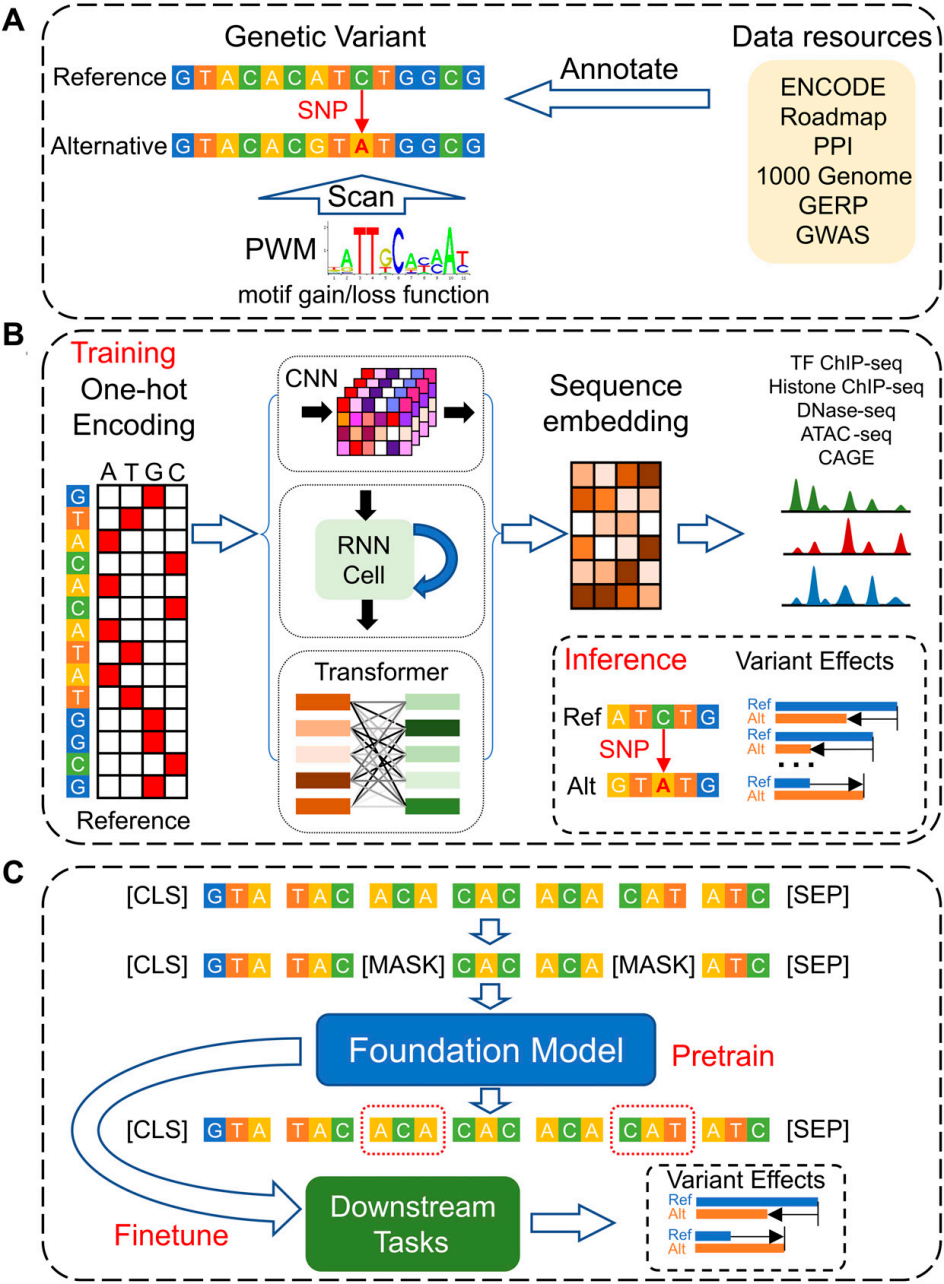
The development of models for genetic variants’ effect predictions based on DNA sequences. **(A)** Traditional models leverage multi-omics data resources to annotate and prioritize genetic variants and use static motif PWMs to analyze the gain- and loss-function of TF bindings. **(B)** Deep Learning models, employing CNN, RNN, and Transformer architectures, are designed to predict functional genomics profiles across various cell types. They determine the effects of genetic variants by comparing the predicted genomic profiles for the reference *versus* alternative alleles. **(C)** Foundation Models utilize a self-supervised pre-training strategy based on DNA sequences only, enabling them to be efficiently fine-tuned for a range of downstream tasks, including the prediction of genetic variant effects across different cellular contexts.

Given the DNA sequence’s fundamental role as the instruction manual for all aspects of life, understanding the function of regulatory genomic elements that control gene expression is paramount. Moving beyond population-based statistical analyses like GWAS and Transcriptome-Wide Association Studies (TWAS) (Wainberg et al., 2019), direct predictions of genetic variant effects from DNA sequences are pivotal for elucidating the underlying biological mechanisms. This review will explore the evolution of computational models for predicting genetic variant effects genome-wide. We first review the traditional annotation-based models that rely on simple sequence motifs to estimate variant impacts, then dive into the advancements achieved through *de novo* prediction models that leverage deep learning techniques (Figure 1). We conclude by discussing the current challenges in the field of systems genetics and proposing future research directions that hold promise for further breakthroughs.

### Functional variant annotation and prioritization

The ENCODE (Luo et al., 2020) and the Roadmap Epigenomics Consortium (Bernstein et al., 2010) have significantly advanced our understanding of the human genome by profiling a wide array of functional noncoding elements through diverse assays. This wealth of data has enabled the functional annotation of genetic variants across the human genome (Figure 1A). GWAVA (Ritchie et al., 2014), by leveraging a comprehensive suite of genomic and epigenomic annotations, predicts the functional impact of noncoding variants. Its features encompass open chromatin regions, TF binding sites, histone modifications, RNA polymerase interactions, CpG islands, genomic segmentation, evolutionary conservation, genic context, and sequence context. These annotations are synthesized to mitigate the challenges posed by context dependency and the variability of evolutionary conservation signals within regulatory elements. Furthermore, pattern recognition algorithms help to identify DNA sequence motifs overrepresented in regulatory regions of co-expressed genes, enhancing our understanding of gene regulation (Stormo and Fields, 1998). The Position Weight Matrix (PWM) (Stormo and Fields, 1998) represents DNA binding sites of different TFs by scoring each potential base at a given genomic position, thereby quantifying the specificity of protein-DNA interactions and facilitating the prediction of new binding sites. An annotation-based approach, Funseq2 (Fu et al., 2014), integrates these methodologies to analyze loss-of-function and gain-of-function events in TF binding. It calculates motif-breaking scores for variants within TF binding motifs identified by ChIP-seq peaks, and motif-gaining scores for variants in promoters or regulatory elements significantly associated with genes, based on PWM *p*-values for the mutated allele. Funseq2 also incorporates annotation-based features such as conservation, enhancer-gene links, network centrality, and recurrence across samples. However, reliance solely on regulatory annotations and static PWMs has its drawbacks: many variants in non-coding regions do not overlap with regulatory annotations, and novel motifs cannot be discovered through static PWMs (Zhou and Troyanskaya, 2015; Kelley et al., 2016).

Addressing these limitations, kmer-SVM (Lee et al., 2011) emerged as a pioneering model for predicting regulatory elements directly from DNA sequences, bypassing the need for existing annotated motifs. It counts the frequencies of various k-mers within a piece of DNA sequence, employing a support vector machine (SVM) trained on these k-mer features to assess the likelihood of a sequence being a functional genomic regulatory element or a tissue-specific enhancer. Gapped k-mers, utilized as features in the gkm-SVM (Ghandi et al., 2014), have further enhanced model accuracy in enhancer identification and TF binding site prediction. Moreover, Delta-SVM (Lee et al., 2015) incorporates the gkm-SVM predictions to assess the disruptive impacts of genetic variants. Despite these advances, the complexity and non-linearity of the underlying regulatory grammar in DNA sequences require further improvements in model performance (Zhou and Troyanskaya, 2015; Kelley et al., 2016).

### De novo prediction of genetic variants’ effects based on deep learning

Deep learning excels in two key capabilities: 1) extracting and representing features, with enhanced flexibility and power, from semi-structured and unstructured data formats, such as texts and images, and 2) approximating various functions effectively through deep layering, with neural networks comprising stacks of linear transformations interspersed with non-linear activations. For the purpose of predicting the effects of genetic variants (Figure 1B), deep learning models typically represent reference DNA sequences using the one-hot encoding (where A = [1,0,0,0], C = [0,1,0,0], G = [0,0,1,0], T = [0,0,0,1], and N = [0,0,0,0]). The input DNA fragments are represented accordingly, $S\in R^{4\times L}$ , where L denotes the DNA sequence length. Feature extraction from these one-hot encoded sequences to produce sequence embeddings typically employs two foundational architectures: the 1D Convolutional Neural Network (CNN) (O'Shea and Nash, 2015) and the Recurrent Neural Network (RNN) (Sherstinsky, 2018), such as Long Short-Term Memory Network (LSTM) (Sherstinsky, 2018).

The CNN architecture focuses on local sequence information, with the initial layer acting as a position-weight matrix, so that the convolution operations are analogous to computing PWM scores across the DNA sequence within each sliding window. Subsequent deep CNN layers capture the non-linear and complex sequence signatures, by utilizing the pooling layers to reduce dimensions after each CNN layer. On the other hand, the LSTM architectures capture sequential dependencies in the genome, by incorporating an internal state that reflects the long-term sequential information. Following these feature representation layers, several fully connected layers are then utilized to generate the final predictions. CNNs, in particular, are adept at learning hierarchical layers of complex, nonlinear patterns without requiring strong prior biological assumptions, thus enabling the discovery of novel sequence motifs and their organizational sequence contexts (Zhou and Troyanskaya, 2015; Kelley et al., 2016; Quang and Xie, 2016).

Pioneering applications of deep neural networks in this field, such as DeepSEA (Zhou and Troyanskaya, 2015) and Basset (Kelley et al., 2016), have demonstrated the significant potential of CNNs for predicting genetic variants’ effects based solely on DNA sequences. DeepSEA leverages a multi-task CNN model to predict TF ChIP-seq, DNase-seq, and histone mark ChIP-seq peaks across a variety of cell types, based on the data from the ENCODE and Roadmap Epigenomics projects. Basset focuses on chromatin accessibility, while DanQ (Quang and Xie, 2016) combines CNN and LSTM to enhance peak profile prediction performance. Trained on the large-scale multi-omics datasets across different cell types from the reference genome, these deep learning models are thus capable of predicting the peak profiles of distinct regulatory factors in a cell-type specific way. For a specific alternative allele of interest, the model’s predictions based on the altered genome sequence are compared to those based on the reference genome. The differences in predictions are then used as indicators of the alternative allele’s functional disruptions under specific cellular contexts, leading to mechanistic hypotheses of its downstream effects in complex human diseases.

Further advancements have seen models like Basenji (Kelley et al., 2018), which employs CNN architectures to predict a wider range of genomic signals, including DNase-seq, histone mark ChIP-seq, and CAGE signals across cell types. By using dilated convolution layers, Basenji is able to capture more contextual information around 32 kb DNA sequence windows, thereby identifying relevant regulatory sequences over a broader scope. Additionally, efforts to understand genetic variant effects have expanded from modeling the genomic and epigenomic levels to predicting target genes’ expressions. For instance, ExPecto (Zhou et al., 2018) predicts the effects on nearby gene expression in a two-stage strategy. First, ExPecto forecasts histone marks, TF, and DNase profiles from DNA sequences, and second, it aggregates the forecasted signals to make predictions of tissue-specific expression. This approach allows for the interpretation of genetic variants’ effects in the dysregulation of nearby genes. Moreover, BPNet (Avsec et al., 2021a) has pushed the boundaries further by predicting base-resolution genomic profiles, utilizing a CNN architecture without pooling layers to achieve the single-base pair resolution predictions.

### Cross-species regulatory information and long-range variant effects

Expanding the training dataset is a well-regarded strategy to enhance the accuracy of deep learning models. While new genome-wide functional genomics profiles grow fast, these new datasets primarily provide information that has already been captured by the model from existing datasets in the human genome. The additional benefits of gathering more functional genomics datasets from additional human genomes may decrease, since the genotypes of different individuals are largely similar. In this context, the quest for significantly different training sequences becomes paramount, with a greater potential to develop and refine more sophisticated and precise models.

An intriguing solution lies in the exploration of non-human species as a reservoir of novel training data. The regulatory DNA sequences of species that are genetically related to humans possess sufficient similarities, enabling the application of machine learning models trained across these diverse genomes. Such cross-species training has the potential to enhance the models’ understanding of regulatory sequence activities. An example of this approach is the expansion of the Basenji model to simultaneously process functional genomic signal tracks from both the mouse and human genomes (Kelley, 2020). This cross-species training strategy has been shown to yield more accurate predictions on the test set of sequences which has not been seen by the model previously, compared to those trained exclusively on data from a single species. This innovative approach underscores the utility of integrating diverse genomic data sources to significantly advance the precision of predictive models in functional genomics.

However, CNNs, the key architecture in previous models, often struggle with the problem of capturing semantic dependencies over long genomic distances due to their focus on localized feature extraction, which is limited by the filter size. Besides, RNNs can learn long-term dependencies but are hampered by issues like vanishing gradients and inefficiency in dealing with long genomic sequences. This limitation is particularly challenging in modeling complex cell-type specific gene regulation, where distal enhancers can influence gene expression over large distances (Lieberman-Aiden et al., 2009; Wang et al., 2021), underscoring the importance in predicting long-range effects of genetic variants. The Transformer model (Vaswani et al., 2017) has demonstrated remarkable success beyond its initial applications in natural language processing and computer vision, increasingly supplanting traditional CNN and RNN-based models across various domains. Its exceptional capability to capture long-range dependencies without relying on recurrent units renders it more scalable and adaptable for handling large datasets. At the heart of the Transformer architecture is the multi-head self-attention mechanism, which efficiently models dependencies between genomic locations, regardless of their distance (Vaswani et al., 2017). This ability allows deeper layers of the model to discern increasingly complex relationships, facilitating the prediction of distal genetic variant effects by capturing interactions between genomic locations separated by considerable distances.

Enformer (Avsec et al., 2021b), a state-of-the-art model leveraging both CNNs and the Transformer architecture, excels in predicting histone marks, TF binding sites, chromatin accessibility, and gene expression across diverse cell types, including those from the genomes of human and mouse. Its design significantly extends the model’s receptive field, enabling the identification of distal regulatory elements up to 100 kb away. This expansive reach allows Enformer to integrate information from all pertinent regions, such as enhancers, thereby enhancing gene expression prediction. Moreover, the model’s attention weights offer greater interpretability, shedding light on the underlying mechanisms of chromatin and gene regulation. With its superior performance of predictions across >5,000 functional genome profiles, including gene expressions, Enformer showcases an unparalleled capacity to forecast both local and distal genetic variant effects. This demonstrates the potential of Transformer-based models in advancing our understanding and prediction of genetic regulations underlying complex traits.

## General sequence grammar of variants learned by foundation models

Traditional deep learning models have achieved impressive results in interpreting functional genomic profiles from DNA sequences through supervised learning, where the models are trained to accurately predict experimental genomic tracks based on the sequence representations. However, this approach necessitates a vast amount of labeled data, constraining the models’ performance and utility in situations where labeled data is scarce. Obtaining high-quality, labeled datasets is often expensive and time-consuming. Moreover, the available data tends to be biased towards certain well-studied cell types with many tracks, neglecting a broad spectrum of cell types yet to be explored. This imbalance results in overrepresented genomic tracks overshadowing the DNA sequence representation, diminishing the efficacy of genomic variant effect prediction in less studied, underrepresented cell types.

In contrast, the development of Foundation Models originally in the fields such as text and image generation illustrates the potential benefits of leveraging context information through a self-supervised pre-training strategy (Devlin et al., 2018; Brown et al., 2020). These models, trained on enormous datasets, have demonstrated capabilities surpassing human performance in certain tasks. The pre-training and fine-tuning framework of Foundation Models involves initial training on vast unlabeled datasets, followed by fine-tuning for specific downstream tasks (Devlin et al., 2018; Brown et al., 2020). Applied to disease genetics studies, this approach entails pre-training models on unlabeled genomic sequences, which are subsequently fine-tuned for specific genomic interpretation tasks (Figure 1C). This methodology not only mitigates the challenges associated with data scarcity and bias but also enhances the model’s ability to understand and predict across a diverse range of cell types and genomic contexts (Ji et al., 2021).

DNABERT (Ji et al., 2021) is a pioneer encoder-based Foundation Model in genetics. It processes DNA sequences by breaking them down into k-mers. For input sequences with lengths up to 512 bp, 15% of k-mers are randomly replaced by a [MASK] token. The Transformer encoder then leverages context information to reconstruct these masked k-mers without additional information. By accurately reconstructing the masked k-mers, DNABERT captures the fundamental grammatical structures of DNA sequences, enabling it to generate meaningful representations for any given sequence. This model has demonstrated remarkable efficacy across numerous downstream applications (Ji et al., 2021), such as promoter identification, TF binding site prediction, and the detection of functional genetic variants. Building on DNABERT’s foundation, subsequent iterations like DNABERT2 (Zhou et al., 2023) and DNABERTS (Zhou et al., 2024) have broadened the scope of Foundation Models to encompass a wider range of species beyond just humans.

The Nucleotide Transformer (Dalla-Torre et al., 2023), an advanced and larger encoder-based Foundation Model, is pre-trained on DNA sequences with over 2.5 billion parameters and can handle sequences up to 6 kb in length. This model has shown remarkable success in a variety of downstream tasks (Dalla-Torre et al., 2023) after fine-tuning, demonstrating the beneficial impacts of both increased model size and the ability to process longer sequences. Beyond the Transformer architecture, HyenaDNA (Nguyen et al., 2023) innovatively extends the contextual reach to up to 1 million tokens at the single nucleotide level through the use of global convolutional filters. This significant enhancement enables the model to effectively leverage long-range chromatin regulation at single base pair resolution. Additionally, HyenaDNA introduces novel downstream adaptation methods, such as a unique soft prompt technique. This approach allows for exceptional downstream results without the necessity of updates to the pre-trained model, thus facilitating the seamless application of the Foundation Model to various tasks, including the prediction of genetic variant effects. This revolution in model design and functionality marks a pivotal advancement in our capacity to understand and interpret complex genetic information.

## Discussions

This review has explored the evolution of models dedicated to predicting the effects of genetic variants using only DNA sequences (Table 1). Enabled by the widespread availability of multi-omics datasets and enhanced computational resources, researchers have transitioned from basic feature annotation and motif recognition to the development of sophisticated deep learning models. These models, trained through both supervised and self-supervised approaches, have progressively achieved more accurate predictions of the genetic variant effects across a variety of cell types.

**TABLE 1:** Summary of computational models.

Tool	Model architecture	Required data	Link
GWAVA	Annotation-based	Experimental annotation	https://www.sanger.ac.uk/tool/gwava/
Funseq2	Annotation + PWM	Experimental annotation + DNA sequence	http://funseq2.gersteinlab.org/
Delta-SVM	SVM	DNA sequence	https://www.beerlab.org/deltasvm/
DeepSEA	CNN	DNA sequence + experiment peaks	https://hb.flatironinstitute.org/deepsea/
Basset	CNN	DNA sequence + experiment peaks	https://github.com/davek44/Basset
DanQ	CNN + LSTM	DNA sequence + experiment peaks	https://github.com/uci-cbcl/DanQ
Basenji	CNN	DNA sequence + experiment signals	https://github.com/calico/basenji
ExPecto	CNN + regression	DNA sequence + experiment signals	https://github.com/FunctionLab/ExPecto
BPNet	CNN	DNA sequence + experiment signals	https://github.com/kundajelab/bpnet/
Basenji2	CNN	DNA sequence + experiment signals	https://github.com/calico/basenji
Enformer	CNN + Transformer	DNA sequence + experiment signals across species	https://github.com/google-deepmind/deepmind-research/tree/master/enformer
DNABERT	Transformer	DNA sequence	https://github.com/jerryji1993/DNABERT
DNABERT2	Transformer	DNA sequence	https://github.com/MAGICS-LAB/DNABERT_2
DNABERTS	Transformer	DNA sequence	https://github.com/MAGICS-LAB/DNABERT_S
The Nucleotide Transformer	Transformer	DNA sequence	https://github.com/instadeepai/nucleotide-transformer
HyenaDNA	Hyena	DNA sequence	https://github.com/HazyResearch/hyena-dna

Despite their advancements, deep learning models for predicting genetic variant effects face two significant challenges: Firstly, model training predominantly relies on labeled data at the cell type level, which limits their capability to discern the functional effects at the single-cell level. With the advent of single-cell sequencing technologies, such as scRNA-seq, scATAC-seq, and scHi-C, there is an influx of data providing detailed insights into gene expression, chromatin accessibility, and regulation at the single-cell level. This type of data, however, tends to be sparse and noisy. Foundation models, pre-trained on the fundamental sequence grammar, exhibit a strong potential for enhancing their performance through fine-tuning with minimal data, addressing the challenge of integrating single-cell level data. Secondly, the training of current models is anchored to the reference genome, neglecting the diversity and frequency of genetic variations across different genotypes. While these models may excel in predicting genetic profiles based on the reference genome, they primarily capture consensus information, which may not accurately represent the actual effects of genetic variants. The discrepancies between the reference and alternative alleles do not fully encapsulate the impact of genetic variants. CRISPR (Korkmaz et al., 2016; Fulco et al., 2019) technology, which elucidates the casual and real effects of genetic variants, offers valuable insights beyond the reference genomic context. The CRISPR-derived data is expected to help to fill the gap between model predictions and biological reality.
